# Beautiful Old Age

**Published:** 1859-04

**Authors:** 


### BEAUTIFUL OLD AGE.

"What a lovely old man he was, so simple and modest." Such is a traveller's testimony of Humbolt in his ninetieth year; a man "whose greatness has not destroyed his nobleness of heart, but nobleness of heart has rendered still greater." The author of "Cosmos" stands out among a million of men in his intelligence, in his age, in his striking physiognomy; the blue bright eye, the "massive forehead, deep, broad, overhanging;" and the heart too, stands out, in even higher relief, than all the others, and the stranger apostrophises, "what a lovely old man!"

Religion makes a man lovely in his age; true and deep science makes a man lovely in age; and so does a real great heart; but the imperfections of our nature, all together fail to do it, too often, when there is not sound bodily health, underlying the whole. It is good health which moulds the features in smiles, which warms up the affections, and mellows the heart with human sympathies; on the other hand, illness corrugates the brow, freezes up the fountains of lovingness and despondency, and fretfulness reign supreme, unless counteracted by high christian principles.

With so much depending on bodily health when gray hairs come upon us, who shall not say that, next to securing a Bible piety, it should be the aim of all who are truly wise, to do what is possible by study, by observation and steady self denial, to maintain all the time, a high state of bodily health.

To grow kindly as age comes on, is to grow in likeness to, and a fit preparation for companionship with angels in the mansions where all is love; but to grow cross and peevish and complaining, by reason of the irritating influences which a diseased and suffering body exercise over the heart, making it a leafless tree, sapless and dry, when it should have boughs bending almost to the earth, with the delicious fruits of a loving nature,—how wide the contrast. Old age with religion and health, and old age with neither, let Humbolt and Voltaire be the representative men; and let every man determine within the hour, which portrait he will sit to, in what mould he shall be cast; forgetting not, that that mould is in process of formation now.

